# Relationships Between Antihypertensive, Glucose‐ and Lipid‐Lowering Medication Adherence, and Demographic and Clinical Characteristics in American Indian Adults With Type 2 Diabetes

**DOI:** 10.1155/jdr/5960974

**Published:** 2026-05-13

**Authors:** Lisa Scarton, Tarah Nelson, Yingwei Yao, Dannielle Branam, Chanler Podany, Anatolia B. Legaspi, Marilyn Aguila, Diana Orozco, Amal Almutairi, Aisha Rabie, Tianze Jiao, Ara Jo, William T. Donahoo, Richard Segal, R. Turner Goins, Spero M. Manson, Diana J. Wilkie

**Affiliations:** ^1^ College of Nursing, University of Florida, Gainesville, Florida, USA, ufl.edu; ^2^ Choctaw Nation Department of Public Health, Choctaw Nation of Oklahoma, Durant Health Administration, Durant, Oklahoma, USA; ^3^ College of Pharmacy, University of Florida, Gainesville, Florida, USA, ufl.edu; ^4^ College of Public Health and Health Professions, University of Florida, Gainesville, Florida, USA, ufl.edu; ^5^ College of Medicine, University of Florida, Gainesville, Florida, USA, ufl.edu; ^6^ College of Health and Human Sciences, Western Carolina University, Cullowhee, North Carolina, USA, wcu.edu; ^7^ Centers for American Indian and Alaska Native Health, University of Colorado Anschutz Medical Campus, Aurora, Colorado, USA, ucdenver.edu

## Abstract

**Objective:**

This study examined the relationships between medication adherence and demographic and clinical characteristics in American Indian adults with Type 2 diabetes who live on tribal lands and receive medication at no cost to them.

**Research Design and Methods:**

From tribal electronic health record data, we constructed a cross‐sectional cohort of 3042 adults with Type 2 diabetes. Tribal citizens of a federally recognized tribe were included if aged ≥ 18 years, had Type 2 diabetes, had ≥ 1 Choctaw Nation of Oklahoma healthcare encounter in 2018, and were on antihypertensive, glucose‐ and lipid‐lowering medications in 2018. Proportion of days covered (PDC) was used to assess medication adherence with a threshold of ≥ 80%.

**Results:**

The cohort mean age was 59.3 ± 11.6 years; the majority were male (52%), married (52%), BMI ≥ 30 (74%), and lived in a rural setting (87%). Overall, 63% of patients were adherent to their medications with a mean PDC across all medication classes of 0.81 ± 0.18. Patients aged > 55 years had a significantly higher PDC across all three medication classes compared with those aged ≤ 55 years (84% vs. 77%, *p* < 0.001). Also, BMI ≥ 30 (*β* = 0.033, *p* < 0.001) or having a comorbid condition (*β* = 0.040, *p* < 0.001) were each associated with higher overall PDC. Whereas insulin use (*β* = −0.023, *p* = 0.001) and rural residence (*β* = −0.020, *p* = 0.03) were associated with lower overall PDC. Additionally, patients with comorbid kidney or heart disease had a higher overall PDC compared with those without these conditions (*p* < 0.001).

**Conclusion:**

In a setting with access to no‐cost medication, nearly two‐thirds of our sample were considered adherent to their medications. Older adults and those with comorbid conditions or BMI ≥ 30 had higher overall adherence to medications, whereas those residing in rural areas or using insulin had lower overall medication adherence.

## 1. Introduction

American Indian communities have a rich cultural heritage and a strong tradition of resilience in the face of adversity. These communities have made significant strides in preventing and managing Type 2 diabetes. One of the key factors in this progress has been the Special Diabetes Program for Indians (SDPI) established by Congress in 1997, which provides funding to tribes, Indian Health Services (IHS), and urban Indian health programs serving over 780,000 American Indian and Alaska Natives people across 35 states [1]. The SDPI has yielded impressive results: The prevalence of diagnosed diabetes among American Indian and Alaska Native people decreased from 15.4% in 2013 [2] to 13.6% in 2019–2021 [3], and diabetes‐related mortality was 2.3 times higher in American Indian and Alaska Native people compared with White people [4]. Building on this momentum, it is essential to continue to address gaps in knowledge, particularly in rural areas where access to healthcare and health education resources may be limited. As we seek to further improve health outcomes for American Indian adults with Type 2 diabetes, it is critical to understand the complex interplay of factors that contribute to medication adherence, which is essential for preventing complications in this population.

To optimize the management of Type 2 diabetes, a multifaceted approach is often employed, combining lifestyle modifications with pharmacological interventions such as antihypertensive, glucose‐ and lipid‐lowering medications. However, there is a notable gap in research regarding the adherence rates to these medications among American Indian adults residing on reservations. Although limited, two studies with small samples found extremely low medication adherence among American Indian patients with Type 2 diabetes; both examined beliefs about diabetes, medication management, and how these beliefs affected adherence to medication, though they did not focus specifically on those living on reservations [5, 6].

American Indian patients living in non‐IHS or tribal settings exhibited lower adherence to diabetes medications when compared with non‐Hispanic White patients in a large cohort study (*n* = 5,831) of nine U.S. commercial integrated health care delivery systems [7]. However, medication adherence related to antihypertensive and lipid‐lowering drugs was not examined and the American Indian sample was a small portion (< 2%) of the cohort. Prescription copay costs have been identified as a health system level barrier and associated with decreased medication adherence in persons receiving commercial or Medicare supplemental prescription benefits [8–11]. An increase of $10 was associated with a decrease of 1.8%–2.6% in adherence [10]. Therefore, the previously published findings may not be applicable to reservation‐dwelling American Indian adults/patients who receive IHS or tribal healthcare services, including medication, at no cost to the patient.

Our study examined relationships between medication adherence and demographic (age, sex, and residence location) and clinical characteristics (body mass index [BMI], insulin use, and comorbidities) in American Indian adults with Type 2 diabetes who live on Tribal lands and receive medication at no cost to them. Based on prior studies, we hypothesized that medication adherence would be positively associated with younger age, male sex, nonrural residence, and lower BMI.

## 2. Research Design and Methods

### 2.1. Design and Sample

We conducted a cross‐sectional cohort study using deidentified tribal electronic health record (EHR) data. Patients in the cohort had at least one Choctaw Nation of Oklahoma (CNO) healthcare encounter (medication fill or office visit) between Jan 1, 2018, to Dec 31, 2018. To ensure a focus on comprehensive cardiometabolic therapy, only patients concurrently prescribed all three classes of medication (antihypertensive, glucose‐ and lipid‐lowering) in 2018 were included (*n* = 3042 of 10,506 eligible individuals). This inclusion criterion was selected because our objective centered on cardiometabolic medication adherence rather than adherence to single‐condition therapies. Cardiometabolic medication adherence is an area where the existing literature remains limited and inconsistent.

Eligible patients were adult tribal citizens (aged ≥ 18 years) of a federally recognized tribe with Type 2 diabetes, identified by International Classification of Diseases (ICD)‐10 codes. Patients with end‐stage renal disease (ESRD) or other types of diabetes were excluded. Patients with ESRD were excluded from the proportion of days covered (PDC) measure due to the potential inaccuracies in capturing adherence to glucose‐lowering medications from the pharmacy claims data, given the frequent dosage adjustments and influence of glucose‐containing dialysate on glycemic control [12]. Consistent with Pharmacy Quality Alliance (PQA) recommendations, patients using insulin were also excluded from the PDC measure because insulin requires frequent dosage adjustments and is often used beyond the recommended 30‐day period [13]. Despite this issue, for insulin users in this cohort, we calculated their PDC on other medications with the caveat that these data should be interpreted cautiously. We received institutional review board approval from the CNO and the University of Florida.

### 2.2. Setting

Our study data were derived from the CNO, the third largest federally recognized American Indian Tribe located in southeastern Oklahoma. The CNO reservation is spread over 11,000 square miles [14], and the Health Services Authority provided more than 1,000,000 health encounters in 2024 [15].

### 2.3. Data Extraction

From the CNO EHR database, CNO staff members extracted the following deidentified data for 2018: demographic (age, sex, and residence location) and clinical characteristics (BMI, insulin use, comorbidities [kidney disease, stroke/vascular disease, heart disease, and cancers]) and pharmacy prescription refill data (glucose‐lowering, lipid‐lowering, and antihypertensive classes).

### 2.4. Study Measures

Medication adherence was evaluated, using the EHR pharmacy dispensing data, for each of the three classes of medications (glucose‐lowering, lipid‐lowering, and antihypertensive). Each patient′s adherence was assessed based on the methodology called PDC, a measure endorsed by the PQA [16]. PDC was calculated by using the days of supply (numerator) divided by the number of days of prescription (denominator) in 2018. We used the TEN‐SPIDERS tool to detail our PDC calculation method (Table 1) [17]. We calculated PDC for antihypertensive, glucose‐lowering, and lipid‐lowering medications individually. We also calculated an overall PDC for each patient as the arithmetic mean of their class PDCs. A PDC of ≥ 80% was considered adherent.

**Table 1:** TEN‐SPIDERS tool: PDC calculation approach [17].

Parameter	Our definition
Threshold	● PDC was analyzed as a continuous variable with a range of 0–1.
	● Threshold: ≥ 80% adherent; < 80% nonadherent
Eligibility criteria for inclusion in sample	●At least one noninsulin glucose‐lowering medication dispensed between 1/1/2018–12/31/2018.
	● ≥180 calendar days of noninsulin glucose‐lowering medication (does not need to be consecutive dispensing) per instance to be included.
	● Excluded noninsulin glucose‐lowering medications prescribed less than < 28 days.
Numerator and denominator	● Numerator: The number of days medication was dispensed in 2018.
	● Denominator: The number of days in 2018 that fall within a dispense window. A dispense window is defined as a sequence of medication dispenses with no gap (time between when a dispense runs out and the next dispense day) of > 365 days.
Survival	● No access to survival data.
Presupply	● Leftover from presupply carries over.
In‐hospital supply	● No access to hospitalization data.
Dosing information	● Medication, dose, quantity, and days supplied were available.
Early refills	● Early refills are assumed to extend coverage, with stockpiled days added to subsequent coverage.
Switching	● Switches between medications of the same class are counted as continuous coverage.

*Note:* Copyright 2025, the T2DM and cardiometabolic control research team.

Demographic variables included age (operationalized as a continuous variable) as recorded on January 1, 2018, sex (categorized as male or female), and residence location (categorized as rural or nonrural) during 2018. Residence location was derived based on the zip codes of patient residence available in patient visit data. We utilized the Federal Office of Rural Health Policy′s (FORHP) definition (2023 version) of rural zip codes to classify each zip code into rural or nonrural. Patients with mixed rural status during the year (1% of the sample) were classified as rural.

Clinical Characteristics included BMI calculated from measured height and weight, insulin use (categorized as yes or no), and comorbidities: stroke or vascular disease (e.g., cerebral atherosclerosis and peripheral vascular disease), kidney disease (e.g., chronic kidney disease [CKD] Stages 1–4), heart disease (e.g., heart failure, angina, and myocardial infarction), and cancer (specifically colorectal, breast, or pancreatic). We used the medians of a patient′s heights and weights recorded in 2018. Insulin use was derived from patient medication record. A patient dispensed at least 30‐day supply of insulin was classified as having used insulin for the year. To determine comorbidities, we searched for ICD‐10 indicators in patients′ diagnoses from 1/1/2017 to 12/31/2018. The coding definition for kidney disease, heart disease, stroke/vascular disease, and cancer can be found in Table S1.

### 2.5. Statistical Analysis

We used descriptive statistics to summarize demographic and clinical characteristics of the cohort. Medication adherence was measured as a continuous PDC with a range of 0 to 1. Pearson correlations between the PDCs were obtained.

The percentage of missing data was low (1%) for this dataset (Table S2). Multivariate imputation by chained equation was used for missing data processing, where inference was performed on *m* = 50 completed datasets and then aggregate using Rubin′s rule [18]. Variables used in imputation included patient demographics (age, sex, and marital status), comorbidities, insulin use, labs and measurements (systolic blood pressure and low‐density lipoprotein [LDL], hemoglobin A1c [A1c], height, and weight), and PDCs. We inspected trace plots as well as metrics including autocorrelations and potential scale reduction factors to confirm the convergence of the imputation algorithm. We inspected the strip plots of imputations versus observed values to confirm the plausibility of the imputed values. Regression analysis was conducted to examine the association between medication adherence and patient demographic variables such as age, sex, and residence location and clinical characteristics such as BMI, insulin use, and comorbidities. We treated PDC as a continuous outcome in our regression analysis. Although PDC is between 0 and 1, linear regression is used since (1) we were focused on association analysis instead of prediction modeling; (2) the sample size was large; (3) there was minimal collinearity (variance inflation factors of 1.1 or lower); and (4) ease of interpretation. For sensitivity analysis, we also performed an inflated beta regression. Statistical significance was set at a Type I error of 0.05.

## 3. Results

### 3.1. Patient Demographics and Clinical Characteristics

We identified 3105 patients with Type 2 diabetes with valid dispense records for all three classes of medications in 2018. Excluding 29 patients with ESRD and 28 patients with other diabetes, our cohort consisted of 3048 patients. We further removed three patients identified as white and three identified as native Hawaiian or other pacific islanders, resulting in a dataset with 3042 patients. The demographic and clinical characteristics of the sample are included in Table 2. The mean age of the sample was 59.3 ± 11.6 years, with a sex distribution approximately balanced between males and females. The mean BMI was 35.3 ± 7.8 kg/m^2^ and heart disease (e.g., angina and myocardial infarction) and kidney disease were the most prevalent comorbidities, affecting 21.5% and 14.8% of patients, respectively. Most patients resided in a rural setting (87.0%).

**Table 2:** Descriptive statistics for demographic and clinical characteristics of the sample.

Variable	Category	Frequency (%)
Sex	Female	1460 (48.0%)
	Male	1582 (52.0%)
Marital status, two unknown	Married	1587 (52.2%)
	Single	546 (18.0%)
	Divorced	455 (15.0%)
	Widow/widower	374 (12.3%)
	Separated	67 (2.2%)
	Never married	11 (0.4%)
Rural residence, 25 unknown	Rural	2624 (87.0%)
	Nonrural	393 (13.0%)
Comorbidities^a^	Kidney	451 (14.8%)
	Stroke/vascular	258 (8.5%)
	Heart	653 (21.5%)
	Cancer	67 (2.2%)
Insulin^b^	Yes	1003 (33.0%)
	No	2039 (67.0%)
**Variable**	**Mean (SD)**	**Median (IQR), range**
Age	59.3 (11.6)	60 (52–68), 21–91
Height (in), 23 unknown	67.0 (3.9)	67 (64–70), 50–80
Weight (lb), 78 unknown	225.9 (54.8)	219 (188–257), 83–545
BMI, 78 unknown	35.3 (7.8)	34.2 (29.9–39.5), 14.8–87.1

Abbreviation: BMI, body mass index.

^a^A condition recorded in a prior year was assumed to be present.

^b^Yes = had insulin for 30 days or more in 2018.

### 3.2. Medication Adherence (PDC)

The mean PDC was 0.83 ± 0.19 for antihypertensive, 0.81 ± 0.20 for glucose‐lowering, and 0.80 ± 0.22 for lipid‐lowering medications, with the overall PDC of 0.81 ± 0.18 (Table 3). The overall PDC was between 0.80 to 1.0 for 63% of the cohort and between 0 and 0.40 for only 4% of the cohort. There were strong positive correlations (i.e., *r* = 0.68, *r* = 0.72, *r* = 0.73, *p* < 0.001) between the PDC across all three medication classes indicating that patients who adhered to one class of medication were likely to adhere to the others. The weakest of the three correlations was between glucose‐lowering and lipid‐lowering medications, and the strongest was between glucose‐lowering and antihypertensive medications.

**Table 3:** Distribution of PDC (continuous and categorized) by medication class and overall.

Class	Mean (SD)	Median (IQR)	*P* *D* *C* ≥ 0.8	0.4 ≤ *P* *D* *C* < 0.8	*P* *D* *C* < 0.4
Antihypertensive	0.83 (0.19)	0.90 (0.74–0.98)	68%	28%	4%
Glucose‐lowering	0.81 (0.20)	0.87 (0.70–0.97)	63%	32%	5%
Lipid‐lowering	0.80 (0.22)	0.88 (0.69–0.97)	63%	29%	8%
Overall	0.81 (0.18)	0.87 (0.73–0.95)	63%	33%	4%

Individuals with a BMI ≥ 30, who were male or who resided in nonrural areas had higher lipid‐lowering PDCs than individuals with a BMI < 30, female, or rural, respectively. (Table 4). Patients aged > 55 years had a significantly higher mean PDC across all three medication classes compared with those aged ≤ 55 years (84% vs. 77%, *p* < 0.001) (Table 4).

**Table 4:** Medication adherence relationships with demographic and clinical characteristics.

Variable	Category	%	Antihypertensive		Glucose‐lowering		Lipid‐lowering		Overall	
			*M* (SD)	*p*	*M* (SD)	*p*	*M* (SD)	*p*	*M* (SD)	*p*
Age	≤ 55	36%	0.79 (0.21)	**< 0.001**	0.77 (0.21)	**< 0.001**	0.75 (0.23)	**< 0.001**	0.77 (0.20)	**< 0.001**
	> 55	64%	0.86 (0.17)		0.83 (0.18)		0.82 (0.20)		0.84 (0.16)	
BMI	< 30	26%	0.82 (0.19)	0.07	0.79 (0.20)	**0.05**	0.78 (0.22)	**0.03**	0.80 (0.18)	**0.03**
	≥ 30	74%	0.84 (0.18)		0.81 (0.19)		0.80 (0.21)		0.82 (0.18)	
Sex	Female	48%	0.83 (0.18)	0.98	0.81 (0.19)	0.72	0.79 (0.22)	**0.01**	0.81 (0.18)	0.25
	Male	52%	0.83 (0.19)		0.81 (0.20)		0.81 (0.21)		0.82 (0.18)	
Residence	Rural	87%	0.83 (0.19)	0.40	0.80 (0.20)	0.16	0.79 (0.22)	**0.001**	0.81 (0.18)	**0.03**
	Nonrural	13%	0.84 (0.18)		0.82 (0.19)		0.83 (0.19)		0.83 (0.17)	
Kidney	Yes	15%	0.86 (0.16)	**< 0.001**	0.84 (0.18)	**< 0.001**	0.84 (0.19)	**< 0.001**	0.85 (0.15)	**< 0.001**
	No	85%	0.83 (0.19)		0.80 (0.20)		0.79 (0.22)		0.81 (0.18)	
Stroke or Vascular	Yes	8%	0.84 (0.15)	0.43	0.82 (0.17)	0.19	0.82 (0.20)	**0.04**	0.83 (0.15)	0.12
	No	92%	0.83 (0.19)		0.81 (0.20)		0.79 (0.22)		0.81 (0.18)	
Heart	Yes	21%	0.86 (0.15)	**< 0.001**	0.84 (0.17)	**< 0.001**	0.84 (0.18)	**< 0.001**	0.85 (0.15)	**< 0.001**
	No	79%	0.82 (0.19)		0.80 (0.20)		0.78 (0.22)		0.80(0.19)	
Cancer	Yes	2%	0.88 (0.15)	0.05	0.85 (0.16)	0.10	0.81 (0.21)	0.70	0.84 (0.16)	0.15
	No	98%	0.83 (0.19)		0.81 (0.20)		0.80 (0.22)		0.81 (0.18)	
Any comorbidity	Yes	36%	0.86 (0.16)	**< 0.001**	0.84 (0.18)	**< 0.001**	0.84 (0.19)	**< 0.001**	0.84 (0.15)	**< 0.001**
	No	64%	0.82 (0.20)		0.79 (0.21)		0.77 (0.23)		0.79 (0.19)	
Insulin	Yes	33%	0.81 (0.19)	**< 0.001**	0.79 (0.19)	**0.02**	0.79 (0.22)	0.06	0.80 (0.18)	**0.004**
	No	67%	0.84 (0.18)		0.81 (0.20)		0.80 (0.22)		0.82 (0.18)	

There were significant associations between comorbidities and PDC. Specifically, patients with comorbid kidney disease or heart disease had a higher overall PDC compared with those without these conditions (Table 4). Additionally, patients with a history of stroke had higher lipid‐lowering PDC compared with those without the condition (Table 4). In general, having one or more comorbidities was associated with higher medication adherence (*p* < 0.001). Taking insulin, on the other hand, was associated with lower PDC in general.

### 3.3. Multiple Regression Analysis

Multiple regression analyses were conducted to examine the relationship between several predictors (sex, insulin, and BMI ≥ 30, rural residence, comorbid condition status, and age) and medication adherence (overall PDC and PDCs for antihypertensive, glucose‐lowering, and lipid‐lowering medications separately). Age over 55 was consistently associated with a 6% higher PDC across the board (*p* < 0.001). Although we focused on contrasting age > 55 and those 55 or younger, we also performed analysis evaluating age as a continuous predictor, finding that a 10‐year increase in age was associated with a 3% higher PDC (*p* < 0.001). Adjusting for other predictors, having a BMI 30 or higher was associated with a PDC 3%–4% higher (*p* < 0.001), whereas having a comorbid condition was associated with a PDC 3%–5% higher (*p* < 0.001). Insulin use was associated with a 2%–3% lower PDC across the board (*p* ≤ 0.01). Male sex and rural residence were associated with a 2% higher (*p* = 0.01) and a 4% lower (*p* = 0.001) PDC for lipid‐lowering medications, respectively. Rural residence was also associated with a 2% lower (*p* = 0.03) overall PDC. For sensitivity analysis, we also performed an inflated beta regression, whose outcomes can be found in Table S3. Although the coefficient estimates differ between the two tables due to difference in scales, the two analyses were consistent with each other in identifying significant predictors, with the only exception being the association of rural residence with the overall PDC, which was marginally significant in Table 5 and not significant in Table S3.

**Table 5:** Predictors of PDC (overall, antihypertensive, glucose‐lowering, and lipid‐lowering).

**Overall PDC**				
Predictor	Estimate	Std error	95% CI	*p*

Age > 55	0.059	0.007	[0.045, 0.073]	< 0.001
Sex = male	0.007	0.006	[−0.005, 0.020]	0.26
BMI ≥ 30	0.033	0.008	[0.018, 0.048]	< 0.001
Rural	−0.020	0.009	[−0.039, −0.002]	0.03
Having comorbid condition	0.040	0.007	[0.027, 0.054]	< 0.001
Insulin	−0.023	0.007	[−0.036, −0.010]	0.001

**Antihypertensive PDC**				
Predictor	Estimate	Std Error	95% CI	*p*

Age > 55	0.063	0.007	[0.048, 0.077]	< 0.001
Sex = Male	0.000	0.007	[‐0.013, 0.013]	0.97
BMI ≥ 30	0.031	0.008	[0.016, 0.047]	< 0.001
Rural	−0.008	0.010	[−0.027, 0.011]	0.41
Having comorbid condition	0.034	0.007	[0.020, 0.048]	< 0.001
Insulin	−0.029	0.007	[−0.043, ‐0.015]	< 0.001

**Glucose-lowering PDC**				
Predictor	Estimate	Std Error	95% CI	*p*

Age > 55	0.058	0.008	[0.043, 0.073]	< 0.001
Sex = male	0.002	0.007	[−0.011, 0.016]	0.74
BMI ≥ 30	0.032	0.008	[0.016, 0.049]	< 0.001
Rural	−0.015	0.010	[−0.035, 0.006]	0.16
Having comorbid condition	0.036	0.008	[0.022, 0.051]	< 0.001
Insulin	−0.021	0.008	[−0.035, ‐0.006]	0.006

Lipid‐lowering PDC				
Predictor	Estimate	Std Error	95% CI	p

Age > 55	0.057	0.008	[0.041, 0.074]	< 0.001
Sex = male	0.019	0.008	[0.004, 0.034]	0.01
BMI ≥ 30	0.035	0.009	[0.018, 0.053]	< 0.001
Rural	−0.038	0.011	[−0.061, −0.016]	0.001
Having comorbid condition	0.051	0.008	[0.035, 0.067]	< 0.001
Insulin	−0.020	0.008	[−0.036, −0.004]	0.01

### 3.4. Subgroup Analysis

**Figure 1:**
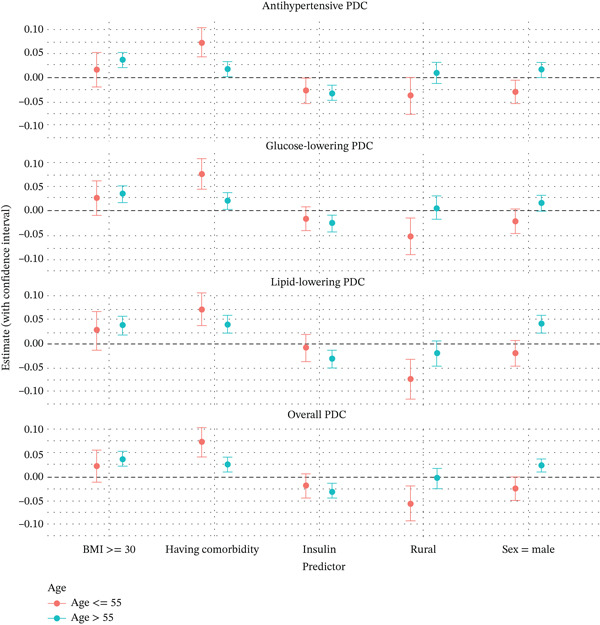
Subgroup analysis of the effects of other demographic and clinical characteristics on medication adherence for patients aged > 55 and ≤ 55.

Given the importance of the age effect, we conducted subgroup analysis examining the effects of other demographic and clinical characteristics on medication adherence for patients aged > 55 and ≤ 55, separately. The estimated effects and corresponding 95% confidence intervals can be found in Figure 1. The moderating effect of age group on the association between medication adherence and BMI or insulin use was not substantial and not statistically significant. On the other hand, we observed that having a comorbid condition was associated with over 7% PDC increase in adherence across the board for patients in the younger age group, but much more modest increase of 2%–4% in PDC for older patients. This interaction was statistically significant for antihypertensive (*p* = 0.002), glucose lowering (*p* = 0.002), and overall PDC (*p* = 0.005), but not for the lipid lowering PDC (*p* = 0.14). Similarly, the rural residence was associated with a lower PDC across the board for younger patients but not clearly associated with a lower PDC among older patients. The interaction was statistically significant for antihypertensive (*p* = 0.02), glucose lowering (*p* = 0.006), lipid lowering (*p* = 0.02), and overall PDC (*p* = 0.007). Previously, we observed sex had minimal or modest association with PDC. Subgroup analysis revealed that among older patients, male sex was associated with higher PDC, but among younger patients, males had somewhat lower PDC than females. The interaction was statistically significant for antihypertensive (*p* = 0.001), glucose lowering (*p* = 0.01), lipid lowering (*p* < 0.001), and overall PDC (*p* < 0.001).

## 4. Discussion

This cross‐sectional cohort of 3042 American Indian adults with Type 2 diabetes receiving care at Choctaw Nation Health Services Authority (CNHSA) is, to the best of our knowledge, the first to examine relationships between medication adherence and demographic and comorbidities among reservation‐dwelling American Indian adults using EHR data. Nearly two‐thirds of patients (63%) were adherent (PDC ≥ 80*%*) to their antihypertensive, glucose‐lowering, and lipid‐lowering therapies. Reasons for adherence in two‐thirds of patients within CNO may include the removal of financial barriers, as medications are provided at no cost; the availability of mail delivery and multiple tribal pharmacy locations; and a strong cultural identity that fosters social cohesion and support. In our study, overall adherence to medications was associated with older age, a higher BMI, and adults with at least one comorbidity. Lower adherence was associated with insulin usage and rural residence.

Our study revealed a slightly higher proportion of adherent patients compared with estimates reported in several meta‐analyses. Adherence to glucose‐lowering medications in our cohort was 63%, compared with 55.5% reported in a meta‐analysis that included both objective and subjective measures and similar to the 62.2% reported in a second meta‐analysis focused on adherence in patients with Type 2 diabetes [19, 20]. Among the patients in our study, adherence was the highest for antihypertensive medications, with a rate of 68% compared with 57% and 60% adherence rates reported in meta‐analyses examining cardiovascular medication adherence in the general population [21, 22]. Additionally, 63% of our patients were adherent to lipid‐lowering medications. This is substantially higher compared with the adherence rate of 49% reported in a recent meta‐analysis of observational studies, though it remains lower than the adherence rate of 90.3% observed in randomized controlled trials [23]. Lemstra et al. [23] identified several risk factors for nonadherence, including new lipid‐lowering use, low socioeconomic status, fewer than two lipid tests performed, the absence of hypertension diagnosis, and use of lipid‐lowering for primary prevention. It is important to note that measures of adherence such as PDC rely on pharmacy data, capturing prescription fill behavior rather than actual medication taking behavior. This limitation may lead to overestimation of adherence, as factors such as medication stockpiling, dose splitting, or intentional noningestion are not detected by fill data alone.

Our findings highlight key demographic factors associated with adherence. Inconsistent with our hypothesis [24], older adults had higher adherence compared with younger adults. Although our findings align with some of the literature [25], cost‐related issues are often cited as a common reason for low adherence among older adults in other settings; whereas patients in our cohort did not have medication‐related co‐pays or out‐of‐pocket costs [25]. Although there was no statistical difference in overall adherence between men and women in our overall analysis, men did have higher adherence to lipid‐lowering medications. Decreased awareness of cardiovascular risk in women and an increase in lipid‐lowering side effects among women may contribute to lower rates of prescriptions and adherence among women [26–28]. Interestingly, in our subgroup analysis, older men (> 55 years) were more adherent overall, whereas younger men (≤ 55 years) were less adherent overall when compared with women. Prior research among American Indian adults with Type 2 diabetes found that gender differences in adherence may be linked to depression or diabetes distress [29]. Possible factors that may explain age or gender differences in adherence should be explored in future studies.

Most participants (87%) lived in rural areas; rural residence was associated with a 2% lower overall adherence as well as 4% lower adherence to lipid‐lowering medications, which was particularly true for younger adults and highlights potential geographic disparities. A systematic review and metanalysis revealed no consistent rural–urban differences in adherence to cardiovascular medications among patients with cardiovascular disease or diabetes [30]. However, it should be noted that there are inconsistencies in how rural and urban settings are defined across studies [30].

Key clinical characteristics were also associated with adherence. Many patients in our cohort (74%) had a BMI ≥ 30, indicating obesity, but contrary to our hypothesis, higher BMI was associated with improved adherence after adjusting for other factors. This finding may reflect the complex relationship between obesity and health behaviors in this population such as an increased perceived need for medication. We found that among patients with comorbidities, the most common conditions were heart and kidney disease. Patients with comorbid heart and kidney disease also had a higher overall PDC compared with those without these conditions. Fifteen percent of the sample had kidney disease, this is substantially lower than 30 to 40 percent of U.S. adults with Type 2 diabetes estimated to have CKD, although patients with ESRD were excluded from our sample [31]. This aligns with the declining rate of ESRD, among American Indian adults likely due in part to prevention programs [32]. The medication adherence gap of patients without a comorbidity compared with those with comorbidities was larger among younger patients. It is important to note that prevalence of comorbidities increase with diabetes duration [33]. Therefore, medication adherence is particularly important for younger adults diagnosed with Type 2 diabetes.

Although our study offers valuable insights, several limitations underscore opportunities for future research and improvement. First, the use of existing EHR data introduces potential concerns regarding data completeness, accuracy, and standardization. Second, the study was conducted among American Indian adults receiving care through the CNHSA, which may limit generalizability to other populations or healthcare systems with differing structures, cultural contexts, or access to care. Third, medication adherence was evaluated using the PDC derived from pharmacy refill data. PDC solely measures medication availability [34] and does not confirm whether medications were taken, potentially overestimating true adherence. Fourth, because only approximately one‐third of all patients with Type 2 diabetes (*n* = 3,046) were prescribed all three medication classes, the study cohort may be comprised of patients with more complex or advanced disease. This potential selection bias could influence the findings as these patients may demonstrate adherence behaviors that differ from those with less intensive therapy or fewer comorbid conditions. To address these limitations, future research should prioritize more comprehensive and culturally relevant approaches. Interventional studies tailored to the unique needs and cultural contexts of American Indian communities are essential to improve medication adherence beyond prescription refills. Additionally, longitudinal studies are needed to elucidate the long‐term effects of adherence on diabetes complications and healthcare costs, addressing the limitations of cross‐sectional designs.

## 5. Conclusion

Our study revealed that nearly two‐thirds (63%) of American Indian adults with Type 2 diabetes, receiving care and no‐cost medications through the CNHSA, were adherent (PDC ≥ 80*%*) to their prescribed antihypertensive, glucose‐lowering, and lipid‐lowering therapies. Factors such as older age, higher BMI, and having a comorbid condition were linked to higher medication adherence, whereas lower adherence was associated with insulin usage and rural residence, suggesting the need for tailored interventions. Among older patients, males had higher PDC, but among younger patients, males had somewhat lower PDC than females. These findings can be used to develop future individual‐level behavioral or system‐level interventions to optimize care in American Indian populations with Type 2 diabetes, including interventions to improve diabetes‐related healthcare programs within CNO.

## Author Contributions

L.S., Y.Y., T.N. and D.J.W. were involved in the conception, design and conduct of the study, and the analysis and interpretation of the results. L.S. wrote the first draft of the manuscript, and all authors edited and reviewed the final version of the manuscript.

## Funding

This research was supported by grant 1R01NR020386‐01 from the National Institute of Nursing Research (NINR), National Institutes of Health

## Disclosure

All authors approved the final version of the manuscript. L.S. is the guarantor of this work and, as such, had full access to all the data in the study and takes responsibility for the integrity of the data and the accuracy of the data analysis. The content is solely the responsibility of the authors and does not necessarily represent the official views of the National Institutes of Health.

## Conflicts of Interest

The authors declare no conflicts of interest.

## Supporting Information

Additional supporting information can be found online in the Supporting Information section.

## Supporting information


**Supporting Information 1** Table S1: ICD‐10 code list.

**Supporting Information 2** Table S2: Missing‐data by variable.

**Supporting Information 3** Table S3: Alternative multiple regression analysis using an inflated beta regression.

## Data Availability

Since we are using deidentified data under an executed data use agreement with Choctaw Nation of Oklahoma, we do not have the authority to share the data.
