# Supplementary material for: Relationships Between Antihypertensive, Glucose‐ and Lipid‐Lowering Medication Adherence, and Demographic and Clinical Characteristics in American Indian Adults With Type 2 Diabetes
Source: J Diabetes Res. 2026 May 13;2026:5960974. doi: 10.1155/jdr/5960974 (PMC13170181; doi:10.1155/jdr/5960974)
Supplement: Supplementary file 1 — Supporting Information 1 Table S1: ICD‐10 code list. [file JDR-2026-5960974-s001.docx]

**Supplemental Table 1: ICD-10 code list**

| icd | label | category |
| --- | --- | --- |
| C18.0 | Malignant neoplasm of cecum | cancer |
| C18.2 | Malignant neoplasm of ascending colon | cancer |
| C18.3 | Malignant neoplasm of hepatic flexure | cancer |
| C18.4 | Malignant neoplasm of transverse colon | cancer |
| C18.6 | Malignant neoplasm of descending colon | cancer |
| C18.7 | Malignant neoplasm of sigmoid colon | cancer |
| C18.8 | Malignant neoplasm of overlapping sites of colon | cancer |
| C18.9 | Malignant neoplasm of colon, unspecified | cancer |
| C20. | Malignant neoplasm of rectum | cancer |
| D12.6 | Benign neoplasm of colon, unspecified | cancer |
| C25.0 | Malignant neoplasm of head of pancreas | cancer |
| C25.3 | Malignant neoplasm of pancreatic duct | cancer |
| C25.7 | Malignant neoplasm of other parts of pancreas | cancer |
| C25.9 | Malignant neoplasm of pancreas, unspecified | cancer |
| C50.019 | Malignant neoplasm of nipple and areola, unspecified | cancer |
| C50.119 | Malignant neoplasm of central portion of female breast, unspecified | cancer |
| C50.419 | Malignant neoplasm of upper-outer quadrant of female breast, unspecified | cancer |
| C50.519 | Malignant neoplasm of lower-outer quadrant of female breast, unspecified | cancer |
| C50.819 | Malignant neoplasm of overlapping sites of female breast, unspecified | cancer |
| C50.919 | Malignant neoplasm of unspecified site of unspecified female breast | cancer |
| C50.929 | Malignant neoplasm of unspecified site of male breast, unspecified | cancer |
| I20.9 | Angina pectoris, unspecified | heart |
| I21.09 | ST elevation (STEMI) myocardial infarction involving other coronary artery of anterior wall | heart |
| I21.3 | ST elevation (STEMI) myocardial infarction of unspecified site | heart |
| I25.10 | Athlerosclerotic heart diease: without hemody | heart |
| I25.2 | Old myocardial infarction | heart |
| I25.84 | Coronary athlerosclerosis due to calcified coronary lesion | heart |
| I25.9 | Chronic ischemic heart disease, unspecified | heart |
| I48.91 | Unspecified atrial fibrilation | heart |
| I50.9 | Heart failure, unspecified | heart |
| I63.9 | Cerebral infarction, unspecified | stroke/vascular |
| I65.23 | Occlusion and stenosis of bilateral carotid arteries | stroke/vascular |
| I65.29 | Occlusion and stenosis of unspecified carotid artery | stroke/vascular |
| I67.2 | Cerebral athlersclerosis | stroke/vascular |
| I67.9 | Cerebrovascular disease, unspecified | stroke/vascular |
| I73.9 | Peripheral vascular disease, unspecified | stroke/vascular |
| N18.1 | Chronic kidney disease, stage 1 | kidney |
| N18.2 | Chronic kidney disease stage 2 (mild) | kidney |
| N18.3 | Chronic kidney disease, stage 3 (moderate) | kidney |
| N18.4 | Chronic kidney disease, stage 4 (severe) | kidney |
| N18.5 | Chronic kidney disease, stage 5 | kidney |
| N18.9 | Chronic kidney disease, unspecified | kidney |
