# Supplementary material for: Relationships Between Antihypertensive, Glucose‐ and Lipid‐Lowering Medication Adherence, and Demographic and Clinical Characteristics in American Indian Adults With Type 2 Diabetes
Source: J Diabetes Res. 2026 May 13;2026:5960974. doi: 10.1155/jdr/5960974 (PMC13170181; doi:10.1155/jdr/5960974)
Supplement: Supplementary file 2 — Supporting Information 2 Table S2: Missing‐data by variable. [file JDR-2026-5960974-s002.docx]

**Supplemental Table 2: Missing-data by variable**

| Variable | % Missing |
| --- | --- |
| Sex | 0% |
| Marital status | 0.1% |
| Age | 0% |
| Height | 0.8% |
| Weight | 2.6% |
| Antihypertensive PDC | 0% |
| Glucose lowering PDC | 0% |
| Lipid lowering PDC | 0% |
| A1c | 5.3% |
| LDL | 14.5% |
| Systolic blood pressure | 1.6% |
| Rural residence | 0.8% |
| Kidney disease | 0% |
| Stoke/vascular disease | 0% |
| Heart disease | 0% |
| Cancer | 0% |
| Insulin use | 0% |
