# Supplementary material for: Relationships Between Antihypertensive, Glucose‐ and Lipid‐Lowering Medication Adherence, and Demographic and Clinical Characteristics in American Indian Adults With Type 2 Diabetes
Source: J Diabetes Res. 2026 May 13;2026:5960974. doi: 10.1155/jdr/5960974 (PMC13170181; doi:10.1155/jdr/5960974)
Supplement: Supplementary file 3 — Supporting Information 3 Table S3: Alternative multiple regression analysis using an inflated beta regression. [file JDR-2026-5960974-s003.docx]

**Supplemental Table 3: Alternative multiple regression analysis using an inflated beta regression**

| **Overall PDC** | | | | |
| --- | --- | --- | --- | --- |
| Predictor | Estimate | Std Error | 95% CI | p |
| Age > 55 | 0.342 | 0.044 | [0.256, 0.429] | <.001 |
| Sex = Male | 0.061 | 0.040 | [-0.016, 0.139] | .12 |
| BMI ≥ 30 | 0.204 | 0.047 | [0.112, 0.297] | <.001 |
| Rural | -0.075 | 0.060 | [-0.193, 0.042] | .21 |
| Having comorbid condition | 0.273 | 0.043 | [0.189, 0.357] | <.001 |
| Insulin | -0.127 | 0.041 | [-0.208, -0.046] | .001 |
| **Antihypertensive PDC** | | | | |
| Predictor | Estimate | Std Error | 95% CI | p |
| Age > 55 | 0.387 | 0.048 | [0.293, 0.481] | <.001 |
| Sex = Male | 0.005 | 0.043 | [-0.079, 0.090] | .90 |
| BMI ≥ 30 | 0.211 | 0.051 | [0.110, 0.312] | <.001 |
| Rural | -0.000 | 0.065 | [-0.127, 0.127] | 1 |
| Having comorbid condition | 0.249 | 0.047 | [0.157, 0.342] | <.001 |
| Insulin | -0.154 | 0.045 | [-0.242, -0.066] | .001 |
| **Glucose-lowering PDC** | | | | |
| Predictor | Estimate | Std Error | 95% CI | p |
| Age > 55 | 0.304 | 0.046 | [0.213, 0.395] | <.001 |
| Sex = Male | 0.032 | 0.042 | [-0.050, 0.114] | .45 |
| BMI ≥ 30 | 0.186 | 0.050 | [0.088, 0.285] | <.001 |
| Rural | -0.050 | 0.064 | [-0.175, 0.076] | .44 |
| Having comorbid condition | 0.207 | 0.046 | [0.116, 0.298] | <.001 |
| Insulin | -0.088 | 0.044 | [-0.174, -0.002] | .045 |
| **Lipid-lowering PDC** | | | | |
| Predictor | Estimate | Std Error | 95% CI | p |
| Age > 55 | 0.272 | 0.047 | [0.179, 0.365] | <.001 |
| Sex = Male | 0.089 | 0.043 | [0.004, 0.173] | .04 |
| BMI ≥ 30 | 0.201 | 0.051 | [0.101, 0.300] | <.001 |
| Rural | -0.180 | 0.065 | [-0.308, -0.052] | .006 |
| Having comorbid condition | 0.288 | 0.047 | [0.196, 0.380] | <.001 |
| Insulin | -0.094 | 0.045 | [-0.183, -0.005] | .04 |
